# Academic Aspects of Lunar Water Resources and Their Relevance to Lunar Protolife

**DOI:** 10.3390/ijms12096051

**Published:** 2011-09-19

**Authors:** Jack Green

**Affiliations:** Department of Geological Sciences, California State University, Long Beach, CA 90840, USA; E-Mail: jgreen3@csulb.edu; Tel.: +1-562-985-4198; Fax: +1-562-985-8638

**Keywords:** fumaroles, lunar water, protolife, lunar volcanism

## Abstract

Water ice has been discovered on the moon by radar backscatter at the North Pole and by spectrometry at the South Pole in the Cabeus crater with an extrapolated volume for both poles of conservatively 10^9^ metric tons. Various exogenic and endogenic sources of this water have been proposed. This paper focuses on endogenic water sources by fumaroles and hot springs in shadowed polar craters. A survey of theoretical and morphological details supports a volcanic model. Release of water and other constituents by defluidization over geological time was intensified in the Hadean Eon (c.a. 4600 to 4000 My). Intensification factors include higher heat flow by now-extinct radionuclides, tidal flexing and higher core temperatures. Lesser gravity would promote deeper bubble nucleation in lunar magmas, slower rise rates of gases and enhanced subsidence of lunar caldera floors. Hadean volcanism would likely have been more intense and regional in nature as opposed to suture-controlled location of calderas in Phanerozoic Benioff-style subduction environments. Seventy-seven morphological, remote sensing and return sample features were categorized into five categories ranging from a volcano-tectonic origin only to impact origin only. Scores for the most logical scenario were 69 to eight in favor of lunar volcanism. Ingredients in the Cabeus plume analysis showed many volcanic fluids and their derivatives plus a large amount of mercury. Mercury-rich fumaroles are well documented on Earth and are virtually absent in cometary gases and solids. There are no mercury anomalies in terrestrial impact craters. Volcanic fluids and their derivatives in lunar shadow can theoretically evolve into protolife. Energy for this evolution can be provided by vent flow charging intensified in the lunar Hadean and by charge separation on freezing fumarolic fluids in shadow. Fischer-Tropsch reactions on hydrothermal clays can yield lipids, polycyclic aromatic hydrocarbons and amino acids. Soluble polyphosphates are available in volcanic fluids as well as vital catalysts such as tungsten. We conclude that the high volume of polar water resources supports the likelihood of lunar volcanism and that lunar volcanism supports the likelihood of protolife.

## 1. Introduction, Water Sources and Defluidization

**Figure 1:**
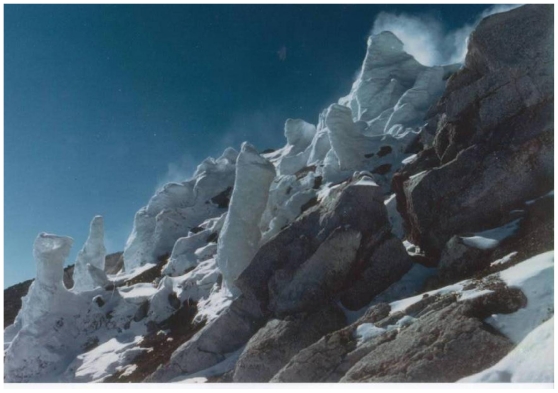
Ice Towers over Fumaroles at the Erebus Caldera, Antarctica. Some spires are over 2 meters high, consisting of ice derived from mostly recycled non-magmatic surface or subsurface waters. Fumarolic activity at Erebus is continuous and intensified during periods of volcanic eruptions (1841, 1900 (?), 1908, 1911, 1912, 1915, 1947, 1955, 1963, and 1972–1984). These dates as shown in the literature have been verified. Photo courtesy of Dr. E. Stump, Arizona State University.

We choose to emphasize the two most important discoveries of water on the moon while acknowledging those of Prospector and Clementine. The first major discovery was by the Chandrayaan 1–mini-SAR mission from mid-February to mid-April, 2009 was based on backscattered radar signals from the north lunar pole, mid-February to mid-April, 2009 (Spudis *et al*.) [1]. Based on information in the Spudis *et al.* article as reported by NASA in March 2010 the volume of water discovered in the lunar north pole using radar backscatter of circular polarization ratios was estimated to be 600 million metric tons of water ice. Circular polarization ratio is defined as the ratio of the power of the received radar signal in the same sense (same sense circular) to that of the opposite sense as transmitted (SS/OS) during the LCROSS mission to the south lunar pole, spectroscopic analysis of the impact plume from the Cabeus crater showed 155 kg of water and other constituents. The LCROSS reference is a lengthy multi-authored section in science, vol. 330, 22 October 2009 listing some 75 authors or co-authors. We extrapolate the total lunar water in inventory of both the north and south poles to be 10^9^ metric tons of water ice. This may be a conservative estimate because the total area of shadowed polar craters of the moon is 1000 square kilometers exclusive of an unknown volume of hydrothermally altered rocks at depth. What is the origin of this water? We believe the water was delivered to the lunar poles by defluidization processes (Rubey) [2] and not by comets. Defluidization is defined as the release of fluids from the interior of a cosmic body to the exterior. In order to elaborate on the fundamental process of defluidization, a crater similar to Cabeus on the Moon is a similar crater Erebus that exists at the south pole of the Earth in Antarctica. A fumarole is a volcanic gas or fluid vent. Low temperature fumaroles may grade into hot springs (LeMasurier and Wade) [3]. The Erebus caldera in Antarctica produced multiple eruptions of the associated fumarole field building up fumarolic spires or towers some 3 meters tall (Figure 1). The ice in these towers would logically consist of isotopically lighter constituents because of multiple cycles of evaporation and precipitation of these lighter constituents. However, carbon dioxide gases emanate from these fumaroles. Wardell *et al.* [4] have analyzed carbon dioxide gases from within the ice towers. The isotopic composition of CO_2_ from these towers measures −2.1 to −4.7 parts per mil and leads to the identification of a magmatic origin of the CO_2_ or a present-day miniature defluidization system. From a larger viewpoint, volcanism is the “skin” effect of defluidization. Was the water delivered to the lunar poles by the impact of meteorites or comets? Water is required for volcanic processes. Water is also required for life processes. The second objective of this article is to show how water would be integral in the evolution of protolife in lunar shadow zones. We define protolife as the evolution of fumarolic fluids into organic molecules via lipids, biofilms, clay/pyrite templates, catalysts such as tungsto-enzymes, and soluble volcanic polyphosphates into pre-RNA molecules, to RNA to self-replicating DNA. These transitions were operative possibly in the Hadean Eon 4.6 to 4 billion years ago, the duration of which closely approximates the entire Phanerozoic Eon on Earth.

Realizing that water is required in all volcanic processes, we examine the following five lines of evidence for lunar volcanism:

1. Theoretical: Tidal and gravity forces intensifying lunar volcanism;
2. Statistical: Distribution of large lunar craters;
3. Chemical: Fugacity and reflectivity of lunar “fire fountain” spheres;
4. Morphological: Endogenic for 77 features of large lunar craters;
5. Geochemical: Concentration of mercury in fumaroles and volcanic plumes.

### 1.1. Theoretical—Tidal and Gravity Forces Intensifying Lunar Volcanism

**Figure 2:**
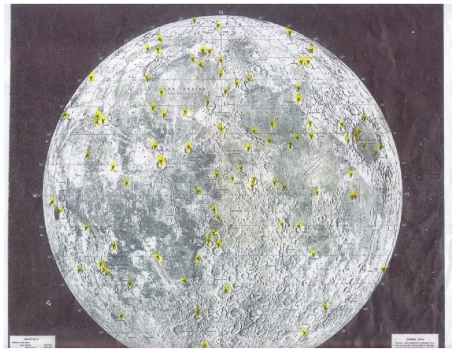
Lunar Transient Sites. Some 2254 changes on the surface of the moon have been reported over the last 500 years. The figure shows the location of some of the transients. Many appear to cluster at the margins of maria. The author believes transients may represent defluidization sites along fractures possibly intensified by tidal flexing. Most reports of transients are in Aristarchus 23.7N, 47.4W. Radon and argon have been reported in fractures associated with Aristarchus which has been interpreted as a “dawn” effect. Cyanogen has been spectroscopically verified at Aristarchus as a transient phenomenon.

**Figure 3:**
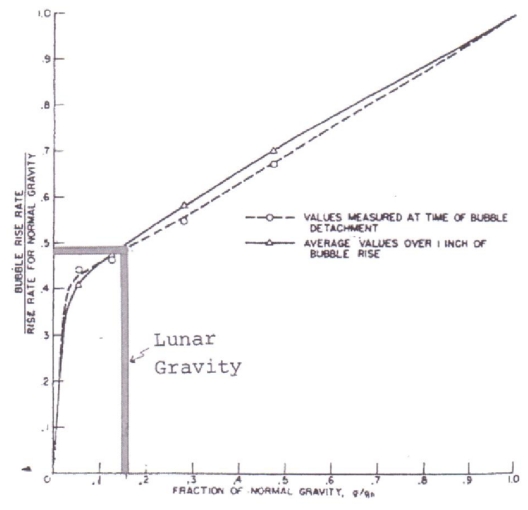
Bubble Rise Rates. Slower rise rate of bubbles under low gravity modified after Usiskin and Siegel [9]. Under lunar gravity, the rise rate of bubbles, all other factors held constant, will be about one half the rise rate of bubbles under Earth gravity. Bubble diameters would also be about 1.7 times larger holding pressure constant.

We elect not to review Green [5] on tidal and gravity processes intensifying lunar volcanism. Tidal and gravity forces may have played a major role intensifying both terrestrial and lunar volcanism during the Hadean Eon as it may today in Io. Realizing the 81.3 to 1 mass ratio of the Earth to the moon, the moon appears to be more rigid than the Earth. The vertical component of the Love number (as a vector pointing to the Earth) shows that the moon is more rigid than the Earth with a K2 number of 0.35 Dickman [6]. Thus, body tides on the moon are as low as 10 centimeters compared with the Earth’s gravitational distortion of some 50 centimeters. However, what is often overlooked is the gravitative pull on fluids. Ocean tides on Earth, for example, greatly exceed 50 centimeters. Likewise, terrestrial gravitative forces on the moon could have mobilized lunar magmas and other fluids which may possibly account for more than 1000 transients of reported changes on the moon (Figure 2). We are aware that most lunar transient data are unreliable because of the hundreds of untrained observers, the multitude of telescope types, and varying weather conditions, eye fatigue and absence of confirmation, except for those which have been spectroscopically verified. In the discussion of lunar transients, the transients may represent release of gases along fractures or fissures. Cyanogen, radon, and argon and carbon dioxide have been spectroscopically recorded. Possibly heavy volatized elements such as mercury could be brought to the lunar surface. In support of this possibility is the high concentration of mercury in the shadowed areas behind lunar rocks where colder temperatures would promote precipitation (von Gunten *et al.*) [7]. This author suggests the mercury is a phenomenon of defluidization possibly associated with transient phenomena over millions of years concentrating in low temperature shadows. Not to be overlooked is the importance of lesser gravity on lunar magma chambers. Figure 3 shows that under lunar gravity, bubble rise rates should be one half of terrestrial rise rates. Further, the slower rise rates of lunar bubbles in lunar magmas may have resulted in enhanced subsidence of lunar Hadean Eon caldera floors creating larger and deeper lunar calderas than on the Earth. We subscribe to the classical definition of a caldera as follows: A caldera is a more or less circular volcanic basin, the diameter of which is greater than the included vents. What is singularly important is that Hadean Eon lunar calderas were regional in distribution as opposed to suture-controlled distribution of volcanic features on the Earth following post Hadean Eon Benioff-style tectonics at convergent and divergent plates as well as at hot spots. The understanding of the difference in Hadean and post Hadean Eon volcanic styles is critical in understanding lunar volcanic mechanisms as they relate to lunar water resources and the possibility of lunar protolife in permanently shadowed regions of the moon, Green [8].

There are two important considerations relative to the slower rise rates of bubbles in lunar magmatic and fumarolic systems. First, there would be enhanced subsidence of lunar craters producing larger and deeper calderas on the moon relative to calderas on Earth. Subsequent eruptions on the floors of lunar calderas in the form of domes and volcanoes would result in these features being lower than the rims of the host caldera on the Earth. The elevations of the internal features on caldera floors on Earth may sometimes be higher than the host caldera rims. Secondly, in a longer bubble-entrained magma or fumarolic column, mixing and convection would be increased favoring reactivity of profolife constituents. Fluidization conditions would also be favored promoting reactivity.

Continuing with the thesis that water is essential for volcanic processes and that water is essential for protolife, we briefly consider a theory on the origin of the moon by Galimov and Krivstov [10]. Their arguments minimize the requirement of an asteroid impacting the Earth to create the moon. Instead, they argue for an asymmetrical dust binary that evolved into the Earth and moon. Positive aspects of the Galimov and Krivstov model are that it accounts for the (1) low iron content of the moon and (2) the integrity of the isotopic ratios of chromium of the Earth and the moon. Their model also documents the volatilization of lunar rock-making elements such as sodium and potassium as well as possible partitioning of a cooler condensing phase in the evolution of the proto-Earth and moon. An important aspect of the Galimov and Krivstov hypothesis is that if an asteroid did impact the Earth to make the moon, it would likely volatilize the lunar water content. The concept of an asteroid creating the moon appears invalid.

### 1.2. Statistical—Distribution of Large Lunar Craters

We turn to item 2 invoking lunar volcanism, namely, statistical. Ronca (1968) [11] has made a statistical evaluation of the distribution of 908 large (greater than 25 kilometers in diameter) lunar craters on the visible side of the moon. He gridded the face of the moon facing the Earth into 172 blocks, each measuring 7.9 × 10^3^ km^2^. Ronca’s results show that the large lunar craters on the Earth-facing hemisphere of the moon based on high confidence limits are not randomly distributed and therefore endogenic (volcanic) in origin. Impact cratering would produce a statistically random distribution but the non-random distribution strongly suggests a volcanic origin of large lunar craters that Ronca included in his determinations.

### 1.3. Chemical—Fugacity and Reflectivity of Lunar Fire Fountain Spheres

The third line of evidence supporting lunar volcanism deals with physio-chemical evidence from the returned “fire-fountain” spheres from the Apollo missions. The spheres clearly contain halogen elements typical of volcanic emanations such as sulfur, chlorine, fluorine and even traces of water. The fire fountain spheres are apparently volcanic vent-related and going back to the first principles in volcanology should be “wetter” than distal vents. A measure of how hydrous magma can be is related to its fugacity or simplistically, oxygen content. Lunar rocks have historically been assigned a fugacity of 10^−13^ atmospheres. Can the spectral reflectance of the lunar spheres be calibrated in terms of fugacity? A portion of the publication by Green 2009 [8] pages 2693–2694 is given below indicating that the fugacities of the Apollo orange and green fire fountain samples can be as high as terrestrial fire fountain spheres of 10^−9^ atmospheres.

Arguments against volcanic or endogenic water are that the majority of large lunar craters are not calderas and that the fugacity or partial pressure of oxygen in lunar melts is too low to permit hydrous environments. Most publications on lunar magmas assume that the oxygen fugacity of terrestrial magmas is 10^−9^ atmospheres. Since oxygen is related to how hydrous a melt might become, the low fugacity of lunar rocks has been a powerful argument against the possibility of “wet” rocks on the moon and hence a water resource. The basis for the claim that lunar rocks are dry is the measurement of fugacity of the returned samples and not of lunar rocks collected at volcanic vent sites. Volcanic rocks at vent sites on Earth are invariably more hydrothermally altered and “wetter” than distal lavas or ash flows. Are all lunar rocks low in oxygen fugacity? Mao *et al.* (1973) [12] performed optical absorbance studies of orange and green glass spherules from the Apollo 17 landing site. Optical absorbance is caused by crystal field and charge transfer processes in iron and titanium in these glasses. When these absorbance data are calibrated by structural parameters based on Fe^57^ Mossbauer resonance measurements, optical absorbance can be used to establish a scale of oxidation or fugacity. Optical absorbance data of synthetic glasses for which the fugacity is known are shown by Mao *et al*. The fugacities of these presumed volcanic vent (fire fountain) spherules are equal to or even greater than most terrestrial volcanic rocks.

### 1.4. Morphological—Endogenic Origin of Morphologic Features

The next to last criterion evaluating the role of lunar volcanism is morphological (4). Appendix 1 tabulates 77 criteria in the following categories:

- General
- Non-mare areas
- Mare areas
- Ray areas
- Remote sensing data
- Returned samples
- Mensuration
- Surface features (<1 km dia.)

In turn, these 77 criteria were scored as follows:

- Volcano-tectonic explanation only;
- Unreasonable by impact;
- Plausible by impact or volcanic processes;
- Unreasonable by volcanic mechanisms;
- Impact explanation only.

Three tabulations are given in Appendix 1.

Where in tabulation A all “c” categories are volcanic,

Where in tabulation B all “c” categories are impact,

In tabulation C (Shown below), we assume that half of the “c” category are volcanic.

**Table:** 

Tabulation C	a plus b	c
I	1	0
II	37	4
III	16	1
IV	8	2
V	2	0
VI	3	1
VII	2	0
Sum	69	8

In Appendix 1 three tabulations are given with tabulation C incorporated into the text. (See legend to appendix) The purpose of these tabulations in Appendix 1 is to assess the importance of volcanic processes on the moon. The tabulations show that volcanism appears to be the dominant surface–shaping mechanism, at least for large craters and vocanotectonic structures. Tabulation A assumes all criteria are volcanic and is considered untenable because it does not include meteorite impact mechanisms. Tabulation B assumes all “C” scores are impact. Tabulation C assumes half of the category are impact and provides a more balanced conclusion to the importance of volcanic processes being the dominant surface-shaping processes on the moon. The term, “apparent dip” under non-mare areas 39 is a geological term referring to the change in tilt angle (dip) as a function of view direction of the tilted strata. References for the 77 entries in Appendix 1 are judgment calls based on 50 years of visiting volcanic provinces around the world and the study of lunar literature. Category I is among the most important categories because it is commonly overlooked. The category is concerned with geological associations; it deals with field relationships. For example, craters associated with internal central volcanoes, domes, ridging, polygonism and, in turn, their relationships with the tectonic environment. What are the relationships of craters with rim craters, lava provinces, and sector grabens? Associations are given an “a” rating.

**Figure 4:**
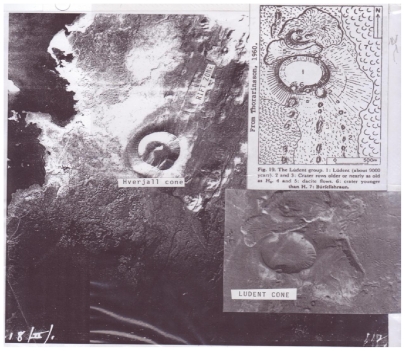
Lùdent and Hverfjall Craters. Area east of Lake Mývatn, north Iceland showing Hverfjall and Lùdent craters and portion of rift region. See text. Image of Lùdent crater courtesy of Landmaelingar Islands.

Explanation of Figure 4 further highlights the significance of associations in Appendix 1. The Mývatn area in northern Iceland is a classical region of lunar-terrestrial analogs. A 100 km portion of the mid-Atlantic ridge fissures is well exposed east of Lake Mývatn which has localized pyroclastic cone craters along a 35 km long segment between the pyroclastic cone of Hverfjall and to the southeast Lùdent crater. These two pyroclastic cones are associated with mid-Atlantic ridge fissures. There are alignments of small basaltic scoria cones as a result of fissure eruptions of 1724 to 1729. Hverfjall, to the northeast of Lùdent, is a 2,500 year old tephra ring with a crater diameter of 1.0 km. The crater is similar to many lunar craters which have central mountains. The hill in Lùdent is 42 meters high. The outward dip of tephra beds varies between 20–42 degrees. Some beds along the southeast interior dip inward.

Lùdent crater is also a pyroclastic cone with a diameter of a kilometer with its rim some 315 meters above its floor. The average dip of the outwardly dipping beds is 21 degrees. The rim is littered with large lava rocks as large as 2 meters. Two rim craterlets exist which do not deform the rim of the parent crater and which are part of 2 crater chains as shown in Figure 4.

Consider the Icelandic pyroclastic cones Lùdent (Figure 4) and the nearby Hverfjall crater. Both Lùdent and Hverfjall are associated with fractures on the mid-Atlantic ridge which define divergent tectonic plates. Both volcanic cones are a few thousand years old and evolved from gas-rich magmas. Lùdent clearly shows felsic lava flows issuing from the northern base of Lùdent. This clear and significant association has been observed by the author in many terrestrial volcanic provinces. First, a subjacent gas-charged magma produces a pyroclastic or cinder cone followed by gas-depleted lava as flows often issuing from the base of the highly vesiculated cone. In the case of the Pisgah cinder cone, California, pahoehoe lava later partially filled the craterlet on the summit of Pisgah crater. Returning to Lùdent crater, there are two rim craters. Both of these rim craters appear to be on partial crater chains. Most significant to lunar analogs is the fact that these rim craters are in sharp contact with the original rim of the parent crater. Cichus crater, a lunar analog of Lùdent crater is 40 km in diameter with a rim crater about 9.6 km wide. Yet, the contact of the rim crater of Cichus with the parent crater is sharp. An impacting meteorite would have destroyed the parent crater rim at its contact with the rim crater. Craters with sharp intersections with the parent crater suggest a volcanic association. Many dozens can be found in lunar craters and are coded “a” in Appendix 1. Cichus is listed in Appendix 2.

### 1.5. Geochemical—Concentration of Mercury in the Cabeus Plume

**Figure 5:**
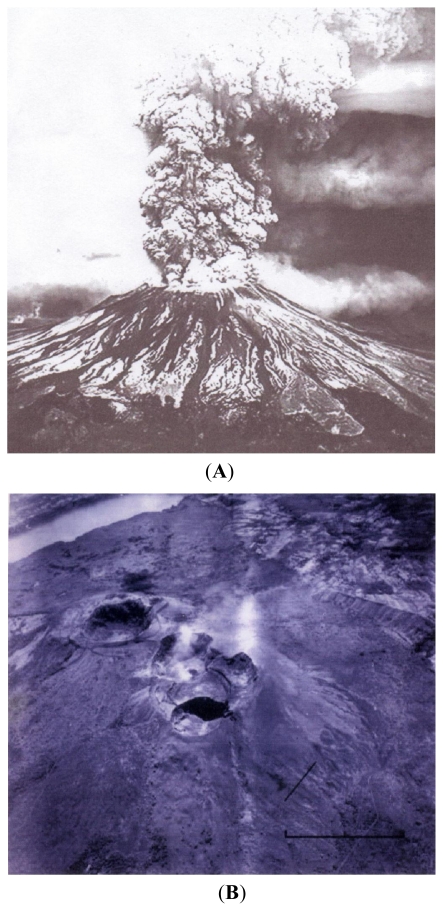
(**A**) 2 March 2010 Eruption of Masaya Caldera, 2N, 86.1W. 11 km diameter multiple vents on the caldera floor on a circular fissure. 4 kilometers in diameter, some of these specific vents may be mercury-rich and the source of much of the mercury cited by Witt *et al*. (**B**) Masaya Caldera, 2N, 86.1W. Multiple vents on the caldera floor sited on a circular fissure, some of which are mercury-rich and the source of much of the mercury cited by Witt *et al.* [14].

The last category of interest in lunar volcanic processes that may account for lunar water resources is (5) Geochemical—a product unexpected in the Cabeus plume was a large quantity of mercury, namely 122 kilograms as reported by Gladstone *et al.* 2010 [13]. Many tens of presumed impact craters have been reported on Earth. No references to high mercury concentrations have been described as present in these craters. Furthermore, the amount of mercury in meteorites is in parts per million with carbonaceous chondrites up to 10 ppm, [14] or slightly more. The amount of mercury in comets including Wild-2 has not been recorded, and is presumably less than 1 ppm. However, mercury is relatively abundant in fumaroles as well documented for Japanese fumarole fields according to Nakagawa [15], who measured the mercury concentrations in the fumarolic gases of twelve geothermal activity areas in Japan. The annual mercury output from these geothermal fumarolic sources in Japan is estimated at 1.4 tons. In 1999, the Masaya caldera in Nicaragua has had a long history of volcanic eruptions (Figure 5). Witt *et al.* [16] measured the mercury output from selected vents in Masaya using Lumex 915+ portable mercury vapor analyzer. The principle of operation of the analyzer is based on the resonance absorption of the 254-nm radiation by mercury atoms vs. a Zeeman correction for nonselective absorption. Particulate mercury was also collected which was 5% of the total gaseous mercury (TGM). The total annual mercury from certain vents in the Masaya caldera is seven tons per year according to Witt *et al*. Another mercury emitter is the La Solfatara fumarole in the Phlegrian fields near Naples, Italy. La Solfatara produces 7 kilograms of mercury per year. Recall that we have previously mentioned there are high concentrations of mercury in the shadow of lunar rocks as reported by von Gunten [7].

The most important conclusion to be drawn from the Witt *et al.* publication is that the mercury found in Cabeus on the moon is most likely of an endogenic or volcanic origin. This concludes part 1 of this paper.

## 2. Part Two—Water Resources and Protolife

As water is essential for volcanic processes, we emphasize that the moon is differentiated and water is required for differentiation, according to O’Hara [17]. Water is also the necessary ingredient for the origin of protolife. Stressed by the author in previous publications are some thought-provoking “coincidences” which may occur in lunar fumaroles given the abundant water that exists there as ices in the permanently polar shadow zones. First, volcanism can provide fumaroles with soluble polyphosphates critical in the evolution of prebiotic organic molecules such as pre-RNA, RNA and DNA. Second, fumarolic activity can provide inorganic templates in the form of hydrothermal products such as pyrite or clay. In addition, not only clay types including fluctuating clay types, some homionic can occur in fumarolic environments. Third, simple combinations of fumarolic gases can create biofilms. Biofilms, consisting of colloidal iron sulfide films on bubbles, would provide both a positively charged energy source. Colloidal iron sulfide as FeS_2_ may increase the production of carbon dioxide by the oxidation of methane. These sulfides as biofilms can also activate amino acids to peptides even after long storage and frigid conditions Heinen [18]. Fourth, the temperature range of hot springs and fumaroles ranging from above 100 degrees centigrade to 50 degrees centigrade is amenable to the survival of protolife. Fifth, sulphur in the Cabeus plume, very abundant in volcanic fluids would be critical in the early evolution of primitive forms of protolife. Sixth, tungsten is more abundant in fumarolic fluids much more so than in Darwin’s “warm little pond” and as tungsto-enzymes Kletzin [19] which are critical catalysts in the evolution of protolife. Seventh, fumarolic environments can broadcast spattered droplets containing concentrated enzymes and catalysts to clay covered distal areas on which the droplets will land. Finally, fumaroles can provide a significant range of hydrous variables within distances of meters from the vent outwards such as temperature, pH, and clay types. This environment, including clay surfaces, is conducive to Fischer-Tropsch reactions capable of yielding lipids, amino acids and polycyclic aromatic hydrocarbons.

Common fumarolic fluids may include water (H_2_O), carbon dioxide (CO_2_), sulfur (S), sulfur dioxide (SO_2_), carbon monoxide (CO), sulfur dichloride (SCl_2_), methane (CH_4_), ethane (C_2_H_6_), carbonyl sulfide (COS), hydrogen sulfide (H_2_S), ammonia (NH_3_), cyanogen (CN), and hydrogen cyanide (HCN) that are of major importance. HCN is fundamental in polymerization reaction creating peptides, polypeptides and nucleosides and in the synthesis of protein ancestors. Matthews and Minard [20] have published on HCN polymerization as a preferred cosmochemical pathway. Other derivative fumarolic fluids can also form when electrically energized; only nano currents may be necessary.

A simple combination of methane and ammonia, for example, produces a critical prebiotic fluid formaldehyde (HCHO) and methanol (CH_4_O). The latter compound was spectroscopically identified in the Cabeus plume. Other combinations of fumarolic fluids may yield ammonium thyocyanate (NH_4_SCN), ammonium cyanide (NH_4_CN), carbon tetrachloride (CCl_4_), acetylene (C_2_H_2_), and ethylene (C_2_H_4_). Isobutene (C_4_H_8_), furane (C_4_H_4_O), benzene (C_6_H_6_) and thiophene (C_4_H_4_S) were measured in fumaroles of the Lascar volcano in Chile.

Of the many primary and derivative fumarolic compounds, many can combine with adequate energy inputs, electrical, shock, *etc*. to form important prebiotic constituents. For example, (1) ammonia and carbon disulfide can form ammonium thiocyanate; (2) Methane plus ammonia can form HCN (hydrogen cyanide). Eutectic freezing of HCN in lunar shadow can concentrate HCN to synthesize nucleic acid bases as well as purines and pyrimidines. Polypeptides can be formed directly from earlier formed HCN; (3) Formaldehyde is an important prebiotic agent and can be formed “in the spark” or by shock waves in a gaseous mixture of water, methane, ammonia, hydrogen and carbon monoxide; (4) Formic acid, a key prebiotic player (HCOOH) is a precursor of lipids in thermocatalytic reactions. Formic acid was the most abundant product in the original Miller experiments; it can also be formed by reacting troilite, the most abundant lunar iron sulfide with hydrogen sulfide and carbon dioxide, the reaction has a negative Gibbs free energy of −11.7 kJ mol^−1^. Another important prebiotic agent is (5), ferrocyanide which can ideally be formed by reacting hydrogen cyanide with ferrous iron to give FE(CN_6_)^4−^. Acetic acid, another prebiotic agent can be formed by reacting methylthiols (CH_3_SH) with CO plus H_2_O to yield CH_3_COOH (Acetic acid), plus H_2_S. Note that clay-armored bubbles of H_2_S occur in the fumaroles of the Uzon caldera in Kamchatka as biofilms which are of significance in the study of the origin of protolife.

There are many stimuli for the origin of lunar protolife in Hadean Eon and later fumaroles. From the vent outwards we see: (1) environmental changes and zonation over distances of meters (eH, pH, freeze/thaw, and fluctuating clay environments including homionic montmorillonite); (2) possible proflavin (PAH) for nucleotide assembly into RNA; (3) flash evaporation; and (4) lower surface pressures. Lower surface pressures would greatly reduce the boiling points of fumarolic fluids and produce larger bubbles extending the vapor phase range of lunar protolife compounds and enhancing reactivity. Reactivity would also be increased by convection and fluidization in fumarolic vents. One of the most interesting stimuli for Precambrian lunar protolife is fumarolic spatter and wet/dry cycles (Green, 2009) [8].

**Figure 6:**
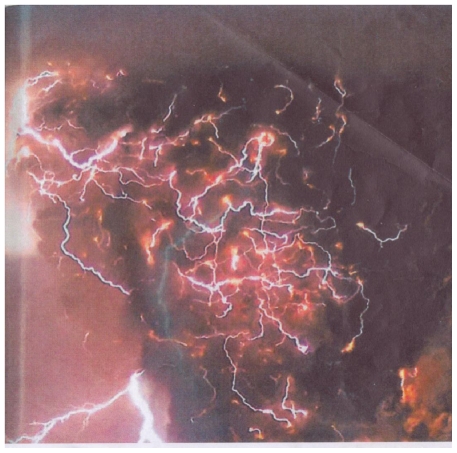
May 2, 2008. Lightning Associated with an Eruption of the El Chaitén Caldera, Chile, 42°50′S 72°39′0. Eruption cloud contains highly charged particles of pumice. (Rhyolitic ash cloud rose to 30,000 meters) Volcanic lightning could have been maximized in the Hadean Eon providing energy for the origin of primary and derivative fumarolic protolife species and other protolife products including cyanogen. Photo credit Carlos Gutierrez, UPI/Digital Railroad.

Fumarolic reactions, many of which occur at negative free energy assure the viability of these chemical reactions. Regarding these reactions, we need to look no further than the Hadean Eon environment which was surely beset with storms of impressive proportions. Lightning is well illustrated in volcanic eruptions today (Figure 6). Volcanic eruptions today produce shock waves which in themselves can create amino acids and possibly peptides in fumarolic fluids. Surface self potentials can also be created pending volcanic eruptions that can conceivably be invoked to mobilize and distribute ash deposits. In permanently shadowed zones at the lunar poles, flow charging potentials can result by freezing volcanic gases. Clathrate structures could possibly result in lunar shadow.

### 2.1. Protolife Evolving

We cite here a presumptuous accounting of steps in the evolution of protolife from fumarolic fluids. In shadow at 40 K, no photodissociation would occur preserving methane and ammonia. Most of the fluids would freeze and be preserved over geologically-long time periods because of their low vapor pressures.

The first evolutionary compounds could include ferrocyanides and soluble polyphosphates. Energy sources in the Hadean Eon such as heat, lightning, sonic shock, electrical potentials produced by charge separation on freezing, could form formaldehyde and hydrogen cyanide. Only microcurrents and nano concentrations are required to form hydroxyl amino acids, the purine bases of the nucleic acid and the pyrimidines bases of the nucleic acids. A second evolutionary stage could episodically concentrate compounds by Fischer-Tropsch catalysis involving hyrodrothermal clays yielding racemic basic and aromatic amino acids and ribose stabilized by boron compounds. Concentration could be increased by freezing fumarolic reflux products and by evaporation and desiccation and by fumarolic spatter. Concentration can also be increased by catalysts and enzymes. A third evolutionary stage might involve polymerization and continued condensation that relate to directed molecular selfassembly on a nanoscale. Organic molecules could be oriented in water monolayers in ice silicate interfaces. Adenine could react with ribose to form adenosine. The latter joining volcanic polyphosphates could produce early forms of adenosine triphosphate or ATP—a metabolic energy source. Then hydrothermal clay templates could assist in the polymerization of amino acids to proteins via peptides and in the creation of bilipid cell membranes of possibly clay-armored vesicles which could withstand the turbulence of fumarolic environments. Iron-sulfur clusters could form in this phase as well as iron sulfide biofilms.

Biofilms consisting of colloidal iron sulfide films on bubbles would provide both a positively charged energy source and a platform to which polar organic molecules could attach and receive energy. Such films are used in present-day flotation operations to separate ore minerals. Colloidal iron sulfide as FeS_2_ may increase the production of carbon dioxide by the oxidation of methane. The sulfides as biofilms can also activate amino acids to peptides. The preceding discussion on protolife evolving is discussed in Green 2009 [8] rigorously substantiated by 146 bibliographic references.

Two new emphases have been published recently relevant to lunar protolife. The first is an increasing role of clay in performing the role of lipids to provide reaction chambers for prebiotic reactions to take place minimizing the dilution from an outside hydrous medium. The second emphasis is on the role of ice itself as the host for prebiotic reactions.

### 2.2. Clay Armored Vesicles

Subramanian *et al.* [21] have described plate-like flakes of montmorillonite collecting on bubbles underwater compartmentalizing organic molecules when clay bubbles come in contact with simple organic liquids such as ethanol and methanol. Both fumarolic fluids are found in fumaroles and some in Cabeus. The organic molecules have a lower surface tension than the water outside resulting in a robust clay armor around the vesicle. The cell is robust enough to withstand the turbulence of fumarolic environments permitting smaller chemical compounds to enter the vesicles but restricting larger structures from leaving.

**Figure 7:**
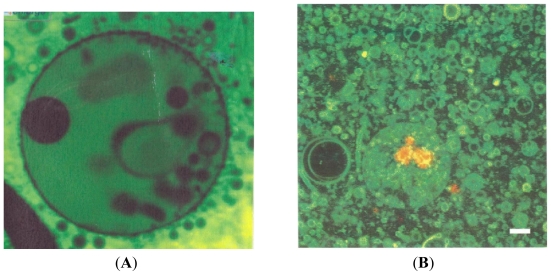
Comparison of a Clay-Armored Vesicle (**A**) with a Lipid Vesicle (**B**). (**A**) Fatty acid liposomes inside a clay-armored vesicle, 100 microns in diameter Photo courtesy of Dr. Amand Abala Subramaniam, Harvard School Engineering and Applied Sciences. (**B**) Red-tagged RNA molecule bound to montmorillonite encapsulated in a non-armored vesicle with a diameter of five microns. Photo courtesy of Dr. M. Hanczyc, Howard Hughes Medical Institute and Department of Molecular Biology, Mass. General Hospital, Boston, Mass. Tessera [20] traces the emergence of vesicles with bilayer membranes composed of a mixture of amphiphilic and hydrophobic molecules and their possible evolution. Bar scale is one micron.

The clay vesicles are covered with small pores exhibiting selective permeability which allows spontaneous compartmentalization of self-assembling molecules in aqueous environments. Figure 7 compares fatty acid liposome compartmentalization within a clay-armored vesicle compared with a non-clay vesicle. In short, clay armored bubbles may have formed the first protocells. The robustness of the vesicles combined with the catalytic properties of montmorillonite may well have had played a role in the creation of protolife in the hydrothermal clay environments of fumaroles. Thus, small fatty acid lipids can enter the clay vesicles and self-assemble into larger structures which then cannot exit. These clay-armored vesicles are semi-permeable. A major highlight of the clay armored vesicles is their robustness to withstand the turbulence of fumarolic environments (Figure 7) (Tessera) [22].

Continuing the discussion of clay-armored vesicles, the montmorillonite collects on the outer surface of air or “fumarolic” (quotes by author) bubbles which may contain simple organic molecules reported in the Cabeus plume. The inner surface also has a lower surface tension than water, the liquid wets the overlying plates of montmorillonite which have catalytic properties and the inner surface of the clay shell becomes wet, the disturbed gas bubble inside would tend to dissolve. The resulting clay vesicle according to Subramaniam is a strong spherical shell that creates a physical boundary that is strong enough to protect their reacting contents from the turbulent environment of fumaroles. The montmorillonite would serve as a catalyst encouraging the enclosed lipids to form membranes of single nucleotides to join into strands of possibly methylated RNA because liposomes and RNA are essential to primordial protolife. Subramaniam and his co-authors suggest that the pores in the clay vesicles could do double duty as both selective entry points and catalytic sites. The most important conclusion drawn (by this author) is the possibility that clay vesicles may be able to evolve by virtue of the catalytic properties of montmorillonite obviating the problem highlighted by Tessera [20]. Because conventional lipid vesicles cannot evolve, clay coated vesicles may possibly evolve more effectively into protolife up to replicating DNA. See Figure 7A.

### 2.3. Ice as a Medium for RNA Evolution

A new study has been published by Attwater *et al.* [23] indicating that protolife may have originated in ice. Water can take on forms near freezing that are loose enough to provide space for the accumulation of unfrozen water and chemical reactants including salts, water and molecules that make up RNA in particular ribozymes, the organic catalyst of RNA. Even at near freezing temperatures, the ribozyme continued to function constructing longer strands of RNA than were constructed at warmer temperatures. However, full strands of RNA were not constructed in Attwater’s test tube experiments. What Attwater *et al.* have shown is that protolife reaction can take place in an ice matrix although Monnard *et al.* [24] has previously shown that biological reactions can take place in ice matrices. Attwater *et al.* argue that ice is a better environment for RNA development than others that have been proposed. RNA is fragile. Its molecular/chemical properties make it relatively easy to disrupt and are probably formed in protected space which Attwater *et al.* believe can be provided for in ice pockets. It is clear that the lunar surface is ideal in affording freezing environments in permanently shadowed zones or intermittently in shadow. This paper strongly suggests that impact exploration of ice deposits on the moon is undesirable and should ideally be made by a properly equipped geologist.

### 2.4. Protolife Overview

The final paragraphs of this paper describe two simple and volumetrically abundant features of fumaroles in lunar shadowed environments: clay and ice. This could be construed simplistically as a consequence of Occam’s Razor, namely that simple products, clay and ice may hold many of the answers in the search for lunar protolife. William of Ockham (1285–1347) originated a principle which gives precedence of simplicity of competing theories: “Entities need not be multiplied beyond necessity.”

We conclude this paper with the obvious—that life on the Earth or protolife on the moon must have had many dead ends during the Hadean Eon. There were many compelling scenes in the Disney movie, *Fantasia*, set to Stravinsky’s “The Rite of Spring” where early life on Earth was destroyed by submarine eruptions several times. Likewise, lunar fumaroles could have erupted periodically especially if blowpiping occurred. Blowpiping can be observed in fumaroles where burning of combustible gases impelled by expanding gases produce blowpiping gases exceeding 400 °C effectively destroying any organic molecule. Dr. R.M. Hazen [25] of the Carnegie Institution compared the multiple destructions of life on Earth to a defective flickering fluorescent lamp.

## 3. Conclusions

Water is necessary for the origins of life. The significance of lunar protolife lies in our ability to prove it. The moon is but a long weekend away. We have developed an array of remote sensors to detect biomarkers. The search for life on Mars, Titan, Europa and Enceladus is clearly beyond on-site human exploration, and the cost of this exploration beyond Mars is orders of magnitude greater than lunar exploration for protolife. The holy grail of science in the search for life may be at our doorstep.

The premise that lunar protolife may have been blown to Earth to trigger life on the Earth is speculative, and which the author deems unlikely. Dynamic lift curves clearly show that volcanic initial ejecta velocities are far lower than the 2.38-kilometers-per-second escape velocity of the moon. Also, it is highly unlikely for hypervelocity ejecta to survive ablation on the journey through Earth’s atmosphere or impact on the Earth. Probably most fragments would be captured in the Lagrangian gravity wells in the moon’s orbit.

Finally, the author presumes with other authorities that the Hadean Eon lunar atmosphere was anoxic. The presumption of lunar protolife is only carried to self-replicating DNA. No oxygen was present to poison protolife evolution. The Earth, however, billions of years later would have evolved to where photosynthesis could have enriched the Earth’s atmosphere with oxygen, a condition not likely in the Hadean Eon on the moon.

Considerable emphasis has been placed on water being integral in volcanic processes reinforced by theoretical aspects of tidal and gravity parameters intensifying lunar volcanisms. We highlight the regional aspects of Hadean Eon volcanism as opposed to suture-controlled volcanism of the post-Hadean Eon. We have touched upon the unlikely aspect of a moon created by asteroid impact of the Earth which would possibly have volatilized the water that now exists on the moon. Statistical treatment of large lunar craters strongly suggests that large craters on the side of the moon facing the Earth are non-randomly distributed reinforcing a volcanogenic origin. The reflectivity of “fire fountain” spheres calibrated into fugacity values suggest the possibility of higher magmatic water concentrations. The moon as a differentiated body would have required water for differentiation. A careful analysis of 77 predominantly morphological features endorses a volcanic origin for large lunar craters, volcanotectonic basins and rayed craters. The impact source of mercury in Cabeus appears unlikely in view of abundant mercury in terrestrial fumaroles, especially in Japan, and the Sulfur Bank Sulfur Mine area, Lake County, California, the Phlegrian Fields, and Masaya. The evolution of protolife was briefly reviewed emphasizing the remarkable coincidences of availability of soluble polyphosphates, biofilms, templates of clay and pyrite, and catalysts. New data on robust clay vesicles and ice pockets were discussed.

Of the main implications of lunar water resources is that water is integral in volcanic processes. As is well known, water will reduce the viscosity of lunar magmas. This fact, low viscosity melts, when coupled with deeper nucleation of bubbles in magmatic melts as a consequence of lesser lunar gravity, would produce a longer bubble-entrained magma column relatively more fluidized than a “drier” melt where reflux and mixing would enhance prebiotic reactions in protolife constituents. We highlight the regional aspects of the distribution of Hadean Eon calderas as opposed to post-Hadean Eon suture-controlled Benoif-style distribution of volcanic structures at plate boundaries and hot spots. We touch upon the unlikely aspects of a moon created by asteroidal impact of the Earth or of a magma ocean which would likely have volatilized the abundant water that now exists on the moon.

Statistical analysis of large lunar craters on the front side of the moon strongly suggests these craters are non-randomly distributed reinforcing their non-impact and hence, volcanic origin. The fugacity of lunar “fire” fountain spheres calibrated into fugacity values yield values of 10^−8^ to 10^−9^ suggest the possibility of higher magmatic water concentrations. A careful analysis of 77 predominantly morphological features endorses a volcanic origin for large lunar craters and volcanotectonic basins including Imbrium, Serenitatis, Orientale and Aitkin and rayed craters. The high amounts of mercury in Cabeus delivered by meteors or comets appears unlikely in view in the mercury deposits in the Phlegrian fields near Naples, Italy, at Mount Etna in Sicily, the fumaroles fields in Japan, at La Soufrière in Guadalupe and in the Masaya caldera in Nicaragua and the relative absence of mercury in terrestrial impact craters and low abundances of mercury in meteorites and in comets.

Armored clay vesicles may have provided the ideal containers providing the compartmentalization of complex organic molecules such as fatty acid liposomes and RNA fragments. However, non-clay armored lipid vesicles cannot evolve. Tessera [22] looks at the possibility of evolving lipid vesicles as a key to the origin of life on Earth. If the clay armor is montmorillionite, the catalytic properties of this clay could permit evolutionary processes to take place leading to the origin of protolife. The montmorillonite armor would be semi-permeable as a catalyst encouraging lipids to form membranes and simple nucleotides to join into strands of RNA. Clay-armored vesicles are robust and can withstand fumarolic turbulence and moreover may have a catalytic montmorillonistic shell. A recent paper deals with a cold start of life with ice as a procellular medium for RNA replication. Previous work had shown that crevices within ice could provide a safe place for the construction of an RNA molecule. As ice forms, pure water becomes crystallized, while salts and other bits of debris accumulate in the watery pockets. These impurities lower the water’s freezing point and the little pockets may remain unfrozen within an otherwise solid chunk. The premise is based on the fact that ice has minute crevices for the construction of RNA molecule ribosomes. According to Attwater *et al.* [21], “a crucial transition in the origin of life is the emergence of a polymer capable of self-repllication and its compartmentalization within protocellular structures. Ice not only promotes the activity of an RNA polymerase ribozyme but also protects it from hydrolytic degradation, enabling the synthesis of exceptionally long replication products. Ice provides quasicellular compartmentalization within the intricate microstructure of the eutectic phase. Eutectic ice phases had been shown to promote the synthesis of nucleotide precursors, as well as the condensation of activated nucleotides into random RNA oligomers. Ice promotes all the steps from prebiotic synthesis to the emergence of RNA self-replication.”

Finally, it is unprecedented to have had a tsuami of ground-breaking new lunar data in the last five years by many scientists in a variety of institutions and governments. We recognize the order that exists in variety, but what of the motivation? Motivation as expressed by Albert Einstein was simply “I want to know.” We have not conclusively explored the moon. The author has in mind some two dozen possible hydrothermal alteration sites that could conceivably contain biomarkers. For example, the interior of the breached central mountain in Copernicus or the summit pits of the dark mounds in Alphonsus at fracture intersections. Lunar exploration has just begun for those “who want to know.” The author had deliberately avoided the practical aspects of lunar water resources such as an international lunar base. There is sizable literature on lunar cement or hydroponics, for example. It does not take a rocket scientist to know how to make fuel from water. We do not have to look too hard to find the L5 Lagrangian Point, or review a tutorial on the Stanford Torus. Recall that the higher the technological level of a program (space or energy), the higher the economical multiplication factor. A lunar exploration/exploitation program should be our goal because we have not yet reached Camelot.

## Appendix I Morphological and Related Features of the Moon

The purpose of this compilation is to evaluate generally operative processes that have shaped the lunar surface: impact of meteorites or volcanism. The scoring of criteria is coded as follows:

**Table:**

Volcano-tectonic explanation only	a
Unreasonable by impact	b
Plausible by impact or volcanic processes	c
Unreasonable by volcanic mechanisms	d
Impact explanation only	e

The following judgmental codes have been assigned to lunar morphological features. Code a is to a volcanic-only origin because no impact mechanism appears impossible. Code b pertains to morphological features that are highly unlikely to be formed by meteoric impact based on studies of terrestrial analogs by the author and interpretations of Clementine orbital and telescopic imagery. Code c includes features that could be formed conceivably by either volcanic or impact processes. The author is aware of tangential ray-like patterns that can be formed by hypervelocity impact as well as similar patterns from volcanic processes. Code d is included for processes deemed unreasonable for volcanic mechanisms. Code e is reserved for impact-only mechanisms.

Eight categories of lunar features are considered:

- General
- Non-mare Areas
- Mare Areas
- Rays
- Remote Sensing Data
- Returned Samples
- Mensuration
- Surface features (<km dia.)

### I. General

**Table:**

Associations	Regional

The category deals with field relationships: Craters associated with internal central mountains, ridging, terracing. In turn, their relationship to a tectonically controlled environment. Interrelationships of craters with rim craters, domes, lava provinces, ash distribution, sector grabens.

### II. Non-Mare Areas

**Table:**

1. Small internally featureless cup-shaped craters within larger craters	Lyot	c
2. Polygonality of craters including (Rectangular and hexagonal)	Ptolemaeus Nicolai area Purbach Barbier Chacornak	a
3. Fault control of crater boundaries	Plato	a
4. Craters perfectly concentric within craters (doubly concentric)	Lavoisier Hesiodus Cassini	a
5. Concentric or partially concentric inner circular mountain ranges within craters	Hertsprung Schrödinger	a
6. Central medial ridges in craters	Alphonsus	a
7. Internal terracing	Bullialdus	c
8. Internal terracing of different ages within craters	Copernicus	a
9. (if not apparent dip) Near horizontal strata on crater walls (apparent dip refers to the change of dip angle where dip is the angle of tilt of layering as a function of view angle. For example, a steeply dipping lava flow might appear nearly horizontal depending on the direction of view.)	Copernicus	a
10. Dark floors of craters	Plato Macrobius Boscovich	b
11. Fractured crater floors	Lavoisier H	a
12. On-ridge or on-fault craters	Bohnenberger	a
13. Leveed sinuous channels or flows leading to crater floors^1^	Copernicus	a
14. Lava-like terrain on crater floors	Tycho Dawes	c
15. Invaded craters on mare “shores”	Fracastorius	b
16. Sector grabens in craters	D’Arrest	a
17. “Pancake” lava flow patterns in craters	Messala border (Orbiter II)	b
18. Rim overturning of craters^2^	None seen by author	a
19. Dark haloed craters many with summit pits within craters	Within Alphonsus Within Dionysius	a
20. Sharp undisturbed overlap of secondary craters on rims of primary or coalesced craters^3^	Cyrillus on Theophilus Harvey on Mach Bordering Cichus Bordering Orontius Bordering Licetus Bordering Barocus Bordering Kozyrev	a
21. Serially overlapping craters	Thebit	a
22. Fused elongate segmented craters	Vogel Torricelli	b
23. Major elongate segmented craters	Schiller	c
24. Tangential alignments	Müller Abulfeda Aristoteles	a
25. Crater chains, some aligned within valleys or grabens and some radial to circular maria	Mairan Sharp Bianchus Condamine Vallis Rheita Vallis Snellius Rima Hyginus Rimae Herigonius	a
26. Curved crater chains	Around Tycho	a
27. Central or near-central mountains within craters	1/3 of all major craters	c
28. Multiple central mountains	Copernicus	b
29. Volcano-like features with summit pits within major craters^4^	Regimantanus Schrödinger Albategnius Lambert	a
30. Central or off-central raised plateaus within craters	Sabine Posidonius	c
31. Central mountains within small craters on or near rims of major craters that lack central mountains	Clavius Mandel’schtam R	a
32. Breached central mountain	Copernicus	a
33. Central craterlet on flat floor within crater	Timocharis	c
34. Major crater-capped domes north of Gruithuisen	Caroline Herschel	a
35. Doming of entire crater floor	Wurzelbauer	a
36. Nested domes within craters	Copernicus	b
37. Dome clusters within craters	Aitken Near NE rim of Gagarin	a
38. Arcuate dome distribution within craters	Zucchius	a
39. Ridged/grooved features on crater floors	SW of Mandel’schtam	b
40. Hummocky terrain surrounding craters	Aristillus Eudoxus	c
41. Outer rim concentric fractures	Archimedes	b
42. Deformed or ruined north and/or south crater margins some with chevron fracture patterns^10^	Arzachel Capella Aristarchus Tycho	

### III. Mare Areas

**Table:**

1. Serial age differences of craters bordering basinal scarps to circular mare “shores”^5^	Rupes Altai to Mare Nectaris	a
2. Antipodal disturbances to circumare basins (Aitken, Orientale, Imbrium)	None seen by author	a
3. Major basaltic dome provinces on crater flanks	SE flank of Copernicus	a
4. Curved valleys (rilles)	Rima Hadley	a
5. Grid fracture pattern	General	b
6. Radial and subradial fractures to circular maria controlling alignment of major exterior craters many with central mountains and some with central mountains with central mountain summit pits	Mare Orientale	a
7. Major interior circumferential features	Mare Orientale Mare Nectaris	c
8. Isolated domes with summit pits (outside of major craters)	North of Hortensius “Schlumberger domes” Near Milchius Near Arago	a
9. Mare ridges localizing craters	Caroline Herschel	a
10. Mascons	Mare Imbrium	b
11. Inward facing scarps within circular maria	Mare Orientale (Rook mountains)	a
12. Flow patterns bordering circular maria	Mare Orientale	b
13. Circular maria^6^	Mare Crisium Mare Orientale Mare Imbrium	c
14. Lava tubes and “skylights”	Near Marius Hills	a
15. Pressure ridges on maria	Serpentine Ridge	b
16. Valleys some with offsets	Vallis Alpine Vallis Triesnecker	a
17. “Ghost” craters within maria	Jansen R	a

### IV. Rays

**Table:**

1. Topographic control of apron-type ray patterns around craters	Kepler	b
2. Loop ray patterns on crater flanks	Copernicus	a
3. Tangential rays	Tycho	c
4. Radial rays	General	c
5. On-ray craters	Bessel	c
6. Elongate craters on rays with axes parallel to regional tectonic grid pattern	SE Copernicus	a
7. Minor craters at ray intersections	Hortensius	b
8. Rays emanating from small rim craterlets or from craterlets exterior to major craters^7^	Copernicus Bergius Sirsalis	b
9. En eschalon structures within rays	N of Copernicus	c
10. “Swirl” ray patterns associated with magnetic anomalies	Mare Ingenii Reiner Gamma Descartes C Gerasimovich D	a

### V. Remote Sensing Data

**Table:**

1. Spectroscopically verified transients	Alphonsus Aristarchus	a
2. Forsteritic (vs. fayalitic) olivine typical of spinifex structures of komatitic lavas on central mountains and terraces^8^	Copernicus	a

### VI. Returned Samples

**Table:**

1. Vesiculated basalt	General	c
2. High fugacity of glass spheres some equivalent to terrestrial fire fountain glass spheres	Apollo 11, 15, 16 and 17 samples	a
3. Halogens in glass spheres similar to terrestrial fire fountain spheres	Apollo 11, 15, 16 and 17 samples	a
4. Breccias	General	c

### VII. Mensuration

**Table:**

1. Non-random distribution of large lunar craters^9^	General	b
2. Diameter to central mountain elevation in large lunar craters	General	a

### VIII. Surface Features Less Than 1 km in Diameter (This category is not included in scoring)

**Table:**

1. Craters produced by primary meteorite impact, by secondary meteorite impact, by volcanic bomb and block impact, by localized regolith sinks, by maar cratering, by ebullition cratering. See 2009, 2010 and 2011 Lunar Reconnaissance Orbiter (LRO) imagery.	General	a to e

1 For II 13 we credit Haffner (1969) for the earliest interpretation of leveed flows in Copernicus as being of volcanic origin.

2 For II 18 a diagnostic criterion for hypervelocity impact, well documented in laboratory experiments, is the creation of overturned strata on impact crater rims. The author has sought but not found ideal examples of crater rim flaps on the moon. Claims for rim overturning on the north and west rims of Aristarchus, in Montes Rook or in Montes Cordillera in Mare Orientalis appear to be unsubstantiated.

3 Relative to II 20, superposed lunar craters are very common. Their coalescing crater rims have sharp contacts as at Parry and Bonpland. Successive overlapping meteorite impacts would destroy or obliterate their intersections. Clementine imagery illustrates such coalescence at a crater called Kozyrev named after a Soviet astronomer who passed away on February 27, 1983. The crater was designated by the International Astronomical Union in 1987 as an impact crater. We disagree. Dr. Nikolay A. Kozyrev was the “dean” of lunar volcanism with his pioneering spectroscopic studies of the endogenic origin of Alphonsus and Aristarchus on the moon. Dr. Kozyrev would be pleased to know and can rest assured that his namesake on the moon is a likely caldera.

4 For II 29, Steinberg (1968) provides detailed descriptions of the Krasheninikov caldera complex and the summit vent on an interior volcano.

5 For III 1, Chabakov (1949) has detailed the slow tectonic evolutionary origin of Mare Nectaris as opposed to its impact origin.

6 Relative to III 13, In the 2010 41st proceedings of the lunar and planetary conference, there was no mention of circular mare formed by volcanotectonic processes nor reference to classical papers dealing with endogenic origins

7 Entry IV 8 deals with radial ray patterns on the moon. The dogmatic treatment of radial rays as splash effects from an impact should be tempered with a sizeable literature on electrostatic and other mobilization mechanisms of lunar rays which are not impact related.

8 For V2, spectral analysis of internal features in Copernicus are claimed to document rebound by meteorite impact of subjacent dunite. Olivine in dunite is usually fayalitic. However, the recorded spectra shows forsteritic olivine typical of near surface spinifex structure of komatitic lavas.

9 For VII 1, see Ronca (1968).

10 For II 42, Green (1965), discussion of possible early Precambrian rotational dynamic effects producing many observed lunar crater marginal distortions is beyond scope of paper.

Tabulation A—Volcanic Model (assuming all “c” categories are volcanic)

**Table:**

	a plus b	c
I	1	0
II	41	0
III	17	0
IV	10	0
V	2	0
VI	4	0
VII	2	0

	77	0

Tabulation B—Impact Model (assuming all “c” categories are impact)

**Table:**

	a plus b	c
I	1	0
II	33	8
III	15	2
IV	6	4
V	2	0
VI	2	2
VII	2	0

	61	16

Tabulation C—Modified Model (assuming half all “c” categories are impact) See Text.

## Appendix II Key to Craters

**Table:**

Name	Location on Moon		Diameter or Length in km	Name	Location on Moon		Diameter or Length in km
1. Aitken	16.9S	173.4E	135	61. Licetus	47.1S	6.7E	74
2. Aitken Basin	56S	180E	2500	62. Linné	27.7N	11.8E	2.4
3. Abulfeda	13.8S	13.9E	65	63. Lyot	49.8S	84.5E	132
4. Albategnius	11.7S	4.3E	114	64. Mach	18.5N	149.3W	180
5. Alphonsus	13.7S	3.2W	108	65. Macrobius	21.3N	46.0E	64
6. Anaxagorus	73.4N	10.1W	50	66. Mairan	41.6N	43.4W	40
7. Arago	6.2N	21.4E	26	67. Mandel’shtam	5.4N	162.4E	197
8. Achimedes	29.7N	4.0W	82	68. Mandel’shtam R	4.5N	159.8E	57
9. Aristarchus	23.7N	47.4W	45	69. Mare Crisium	17.0N	59.1E	555 †
10. Aristillus	33.9N	1.2E	55	70. Mare Imbrium	32.8N	15.6W	1145 †
11. Aristoteles	50.2N	17.4E	87	71. Mare Nectaris	15.2S	35.5E	333 †
12. Arzachel	18.2S	1.9W	96	72. Mare Nubium	21.3S	16.6W	715 †
13. Barbier	23.8S	157.9E	66	73. Mare Orientale	19.4S	92.8W	900 †
14. Barocius	44.9S	16.8E	82	74. Mare Tranquillitis	8.5N	31.4E	873
15. Bessel	21.8N	17.9E	16	75. Marius Hills	14N	56W	14
16. Bianchini	48.7N	34.3W	38	76. Maurolycus	42.0S	14.0E	114
17. Bohnenberger	13.2N	40.0E	33	77. Messala	39.2N	60.5E	125
18. Bonpland	8.3S	17.4W	60	78. Milichius	10.0N	30.2W	13
19. Boscovich	9.8N	11.1E	46	79. Montes Cordillera	17.5S	81.6W	945
20. Bollialdus	20.7S	22.2W	60	80. Montes Rook	17.5S	81.6W	791
21. Byrgius	24.7S	65.3W	87	81. Müller	7.6S	2.1E	24
22. Cabeus	84.9S	35.5W	98	82. Nicolai	42.4S	25.9E	42
23. Caroline Herschel	34.5N	31.2W	13	83. Orontius	40.6S	4.6W	105
24. Cassini	40.2N	4.6E	56	84. Parry	7.9S	15.8W	48
25. Catena Davy	11.8S	8.1W	34	85. Piccolomini	29.7S	32.2E	87
26. Catharina	18.1S	23.4E	104	86. Pitatis	29.9S	13.5W	106
27. Chacornac	29.8N	31.7E	51	87. Plato	51.6S	9.4W	109
28. Cichus	33.3S	21.1W	40	88. Posidonius	31.8N	29.9E	95
29. Clavius	58.8S	14.1W	245	89. Ptolemaeus	9.3S	1.9W	164
30. Copernicus	9.7N	30.1W	93	90. Purbach	25.5S	2.3W	115
31. Cyrillus	13.2S	24.0E	98	91. Pytheas	20.5N	20.6W	20
32. D’Arrest	2.3N	14.7E	30	92. Regiomontanus A	28.0S	0.6W	6
33. Dionysius	2.8N	17.3E	18	93. Reiner Gamma	7.5N	59.0W	70
34. Eratosthenes	14.5N	11.3W	58	94. Reinhold	3.3N	22.8W	48
35. Eudoxus	44.3N	16.3E	67	95. Rhaeticus	0.0N	4.9E	1.6
36. Fernelius	38.1S	4.9E	65	96. Rima Hadley	2.5N	3E	80 *
37. Flamsteed	4.5S	44.3W	21	97. Rima Hygenus	7.4N	7.8E	219 *
38. Fracastorius	21.5S	33.2E	112	98. Rima Sirsalis	15.7S	61.7W	426 *
39. Gargarin	20.2S	149.2E	265	99. Rima Triesnecker	4.3N	4.6E	215 *
40. Goddard A	14.8N	89.0E	89	100. Rimae Herigonius	13.0S	37.0W	100*
41. Guericke	11.5S	14.1W	63	101. Rupes Altai	24.3S	22.6E	427 *
42. Harvey	19.5N	146.5W	60	102. Rupes Recta	22.1S	7.8W	110 *
43. Hercules	46.7N	39.1E	69	103. Sabine	1.4N	20.1E	30
44. Herodotus	23.2N	49.7W	35	104. Schiller	51.9S	39.0W	180 *
45. Hertzsprung	2.6N	129.2W	591	105. Schrödinger	75.0S	132.4E	312
46. Hesiodus A	30.1S	17.0W	15	106. Serpentine Ridge	25N	25E	500
47. Hippalus A	23.8S	32.8W	8	107. Sharp	45.7N	40.2W	40
48. Hortensius	6.5N	28.0E	15	108. Sirsalis	12.5S	60.4W	42
49. Jansen R	15.2N	28.8E	25	109. Sommering	0.1N	7.5W	28
50. Janssen	44.9S	41.5E	190	110. Thebit	22.0S	4.0W	56
51. Kepler	8.1N	38.0W	32	111. Theophilis	11.4S	26.4E	110
52. Kozyrev	46.8S	129.3E	65	112. Timocharis	26.7N	13.1W	34
53. Loa Condamine	53.4N	28.2W	37	113. Torricelli	4.6S	28.5E	23
54. Lacroix	37.9S	59.0W	38	114. Tycho	43.4S	11.1W	102
55. Lambert	25.8N	21.0W	30	115. Vallis Alpes	48.5N	3.2E	166 *
56. Langrenus	8.9S	61.1E	127	116. Vallis Rheita	42.5S	51.5E	450 *
57. Lassell	15.5S	7.9W	23	117. Vallis Schroteri	26.2N	50.8W	168 *
58. Lavoisier	38.2N	81.2W	70	118. Vallis Snellius	31.1S	56.0E	592 *
59. Lavoisier C	35.5N	76.7W	35	119. Vogel	15.1S	5.9E	27
60. Lavoisier T	36.5N	76.6W	19	120. Wood’s Spot	26.4N	50.4W	230
				121. Wurzelbauer	33.9S	15.9W	88
				122. Zucchius	61.4S	50.3W	64

† Diameter of Ring

* Length
